# Administration of combined spinal epidural anesthesia with ultrasound-assisted positioning in obese patients undergoing open hysterectomy: A randomized controlled trial

**DOI:** 10.1097/MD.0000000000036695

**Published:** 2023-12-29

**Authors:** Haihong Yang, Qin Zhang, Zuling Zhong, Yangyang Sun, Huaqu Gong, Yinghai Liu, Xuemei Dai, Lu Lin, Jingya Luo, Gu Gong, Yongjian Yang

**Affiliations:** a Department of Anesthesiology, The General Hospital of Western Theater Command, Chengdu, Sichuan, China; b Department of Anesthesiology, No.950 Hospital, Yecheng, Xinjiang, China; c College of Medicine, Southwest Jiaotong University, Chengdu, Sichuan, China; d Outpatient department, The General Hospital of Western Theater Command, Chengdu, Sichuan, China; e Department of Cardiology, The General Hospital of Western Theater Command, Chengdu, Sichuan, China.

**Keywords:** combined spinal epidural anesthesia, landmark-guided, obese, ultrasound-assisted

## Abstract

**Background:**

Administration of combined spinal epidural anesthesia (CSEA) with traditional landmark-guided positioning can be challenging in patients with high body mass index (BMI). The popularization of ultrasound technology may effectively solve these problems. However, reports on the administration of CSEA ultrasound-assisted positioning in obese populations are relatively limited and have made inconsistent conclusions. We aimed to investigate the ability of ultrasound-assisted positioning to improve the success rate of CSEA in obese patients.

**Methods:**

Overall, 118 adult women with a BMI ≥ 30 kg/m^2^ who scheduled to undergo open hysterectomy and received CSEA were recruited. Finally, 108 patients were enrolled and randomly assigned to 2 groups: the ultrasound-assisted positioning group (group A) and traditional landmark-guided positioning group (group B). Ultrasound-assisted or landmark-guided positioning was employed to locate the puncture interspace before anesthesia. The primary outcomes were the success rate of first attempt and number of attempts. The secondary outcomes were the patient positioning accuracy, positioning time, CSEA operation time, patient-satisfaction scores, anesthesia characteristics, and complications of CSEA.

**Results:**

The success rate of patient first puncture attempt in group A was significantly higher than that in group B (78.4% vs 52.9%, *P* = .007). The total number of punctures was lower in group A than that in groups B (average rank 44.54 vs 58.46, *P* = .005). Using ultrasound positioning as the gold standard, the accuracy of landmark-guided location was only 67%. Positioning time in croup A was longer in group A than that in group B (*P* = .004), while CSEA operation time spent in Group A was less than that in Group B (*P* < .001). Patient satisfaction score in group A was significantly higher than that in group B (*P* = .002). The successful puncture interspace in group A were more likely at L3-4 than that in group B (*P* = .02).

**Conclusion:**

The success rate of first puncture attempt and positioning accuracy in CSEA with ultrasound-assisted is significantly higher than those based on landmark-guided location in obese patients.

## 1. Introduction

Advantages of Combined spinal-epidural anesthesia (CSEA) are fast onset, precise block, and time controllable, and is widely used in the majority of secondary hospitals.^[1]^ Spinal anesthesia could reduce the consumption of local anesthetic, and catheter was used to ensure an adequate block level and mitigation of postoperative analgesia.^[2,3]^ However, CSEA may be challenging when faced with patients in obesity, advanced age, adjacent segment degeneration, or reduced spinal mobility.^[4–6]^ In patients with predictable difficulty of spinal anatomy, especially obese, the failure rate of spinal anesthesia or CSEA is as high as 17%.^[7–9]^

Traditional landmark-guided method is often faced with inaccurate positioning.^[10,11]^ Adult spinal cord mostly terminates at L1 bottom, some may reach L2.^[12,13]^ If the L3-4 interspace cannot be accurately located, there will be a potential risk of accidental injury to the spinal cord by puncturing the subarachnoid space with the intracanal needle when the location is cephalad to the intended interspace. Landmark-guided positioning was based on spinous process and the horizontal line (Tuffier line) connecting the superior points of the bilateral iliac crests, which was assumed to cross the spine at the L3-4 interspace or L4 spinous process.^[14,15]^ However, reliability and accuracy of identifying the spinal level by manually palpating the landmark remains in question.^[16–18]^ In addition, inaccurate positioning and repeated puncture may cause bleeding, infection, and increase patient discomfort and dissatisfaction. Therefore, traditional CSEA techniques need to be optimized to meets obesity challenges and the inherent requirements of enhanced recovery after surgery.

Recently, popularization of ultrasonography has brought about the innovation of anesthesia technology and has emerged as a way to facilitate CSEA perform. Currently, ultrasound has also been likened to clinician second stethoscope, namely operations can be performed under visualization. Studies have shown that pre-puncture or real-time ultrasound scanning, visual identification and marking of the puncture position, can improve the success rate of puncture, shorten the puncture time, reduce the number of needle insertions in predictable anatomical difficulties.^[19–21]^ However, some studies hold inconsistent opinion.^[22,23]^ It shown no benefits with ultrasound-assisted positioning and “inexpert palpation” might exaggerate the benefits of ultrasound.^[24]^ The different conclusions drawn from these studies may be due to the diverse subpopulations, specific circumstances, and certain settings. Thus, it needs to further define the role of ultrasound in the common obese populations, anatomical difficulties or obstetric settings. And this study aims to compare ultrasound-assisted with landmark-guided positioning in terms of the success rate of first puncture attempt, total number of punctures, positioning accuracy and so on in obese patients who underwent open hysterectomy.

## 2. Materials and methods

### 2.1. Study design and patient population

This study was approved by the institutional review board of the No.950 hospital (No: 2021ky003), and written informed consent was obtained from all participating patients. This study was conducted in the No.950 hospital from April 2021 to May 2022, accordance with the Declaration of Helsinki of 1975, as revised in 2013. This trial was registered with the Chinese Clinical Trial Registry (ChiCTR2200058941), and a total of 118 patients were recruited. Trial reporting was in alignment with the consolidated standards of reporting trials 2010 guidelines.

**Figure 1.**
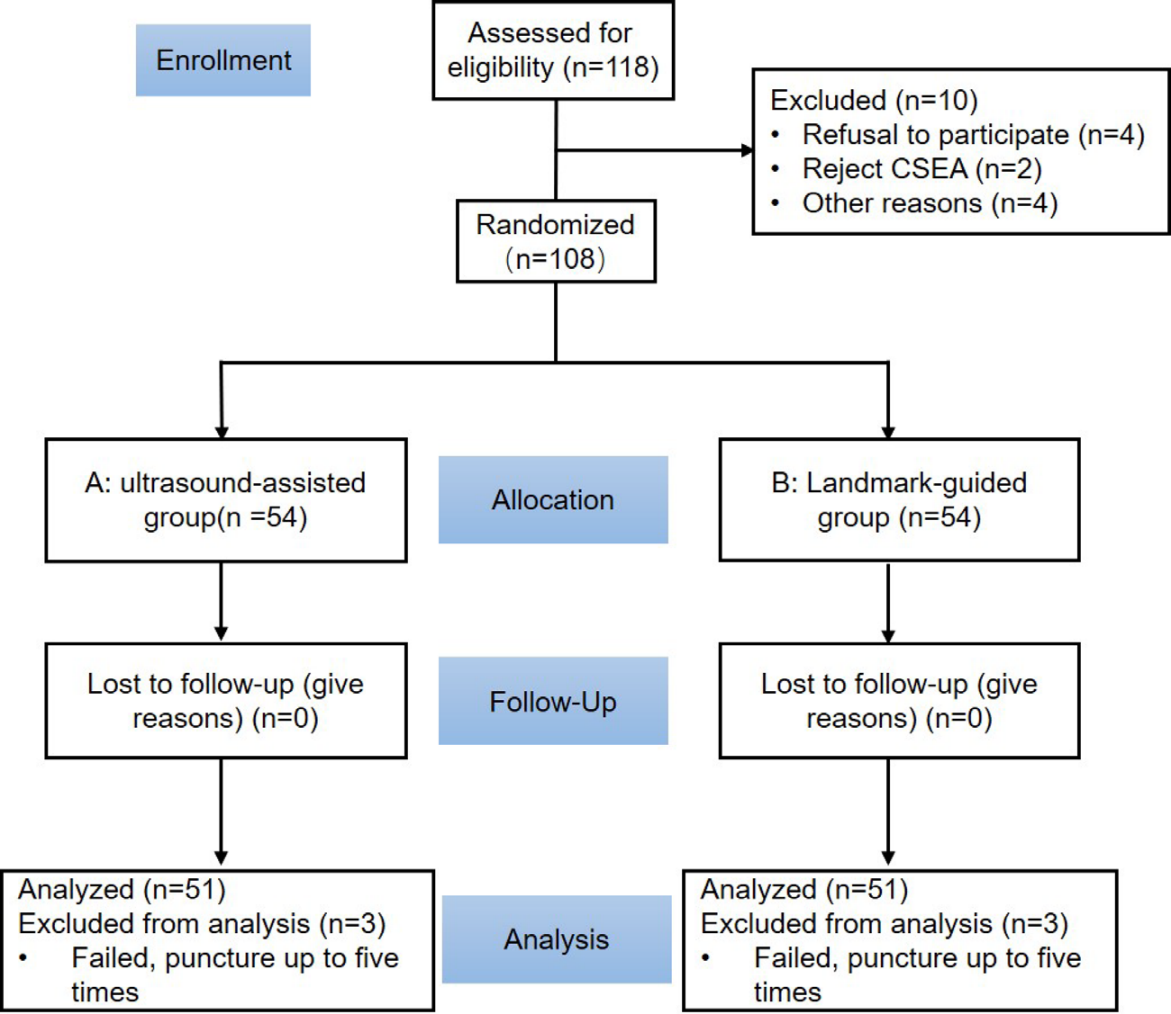
CONSORT flow diagram. CONSORT = consolidated standards of reporting trials.

The inclusion criteria were the following: adult women scheduled for open hysterectomy with CSEA; aged 35~75 years; body mass index (BMI) ≥ 30 kg/m^2^; American Society of Anesthesiologists (ASA) physical status II to III. The exclusion criteria were the following: patients’ refusal to participate; severe cardiopulmonary diseases; contraindications of spinal anesthesia; allergic to local anesthetics. Patients were randomized to ultrasound-assisted group (A) or traditional Landmark-guided group (B) using a computer-generated randomization sequence. According to puncture approaches, 54 patients were divided into traditional group and ultrasound-assisted group respectively (Fig. 1). The same group of senior anesthesiologists (obtaining the title of attending physician; more than 3 years of clinical experience in CSEA with ultrasound) was selected as the operators.

### 2.2. Anesthesia and operating procedures

**Figure 2.**
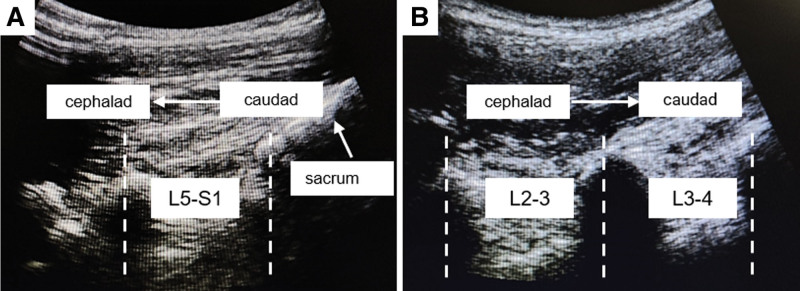
Ultrasound-assisted positioning. (A) The sacrum and L5-S1 interspace were positioned using sliding and tilting scanning techniques in paramedian sagittal oblique orientation with a curved array transducer and the L4-5, L3-4, and L2-3 interspace was successively located by moving the transducer cephalad. Arrowhead indicated sacrum. (B) The T12 vertebra was identified at its joint with the 12th rib and the transducer was moved caudad to identify each consecutive interspace.

All patients received CSEA for open hysterectomy. L3-4 interspace was the first puncture attempt for CSEA, and the L2-3 interspace was chosen as the alternation when the L3-4 interspace puncture was failed. Unless for special reasons, each patient was placed in the right-side position with the back arched and the arms embracing the knees, which facilitated the use of ultrasound by right-handed operators. In group A, the needle puncture location was marked by ultrasound guidance that was administered using a Sonosite Edge portable system. The puncture interspace was confirmed through twice ultrasound scans. At the first confirmation (Fig. 2A), the sacrum and L5-S1 interspace were positioned using sliding and tilting scanning techniques in paramedian sagittal oblique orientation with a curved array transducer. The L4-5, L3-4, and L2-3 interspace was successively located by moving the transducer cephalad, and each point was timely marked on the midline of spine. For the second confirmation (Fig. 2B), T12 vertebra was identified at its joint with the 12th rib. The transducer was then moved caudad to identify each consecutive interspace descending to L4-5. On ultrasound images, the longest transverse process is the L3 lumbar vertebra. The L4-5, L3-4, and L2-3 interspace was reconfirmed, and a new mark was made on the skin. In group B, traditional landmark-guided positioning was used, and the L3-4, L2-3, L4-5 interspace was marked according to the bony markers. The horizontal line connecting the highest points of iliac crests was assumed to cross the spine at the L3-L4 interspace or L4 spinous process. According to palpation of the spinous process, the intervertebral space was determined finally. When the CSEA operation was completed in group B, the ultrasound-assisted positioning was used to locate the actual intervertebral space of L3-4. This was not repeatedly counted into the landmark-guided positioning time.

After positioning the puncture interspace, disinfection and drape were performed. Local anesthesia was administrated with 1% lidocaine and CSEA operation was conducted aseptically throughout the process. Negative pressure method was used to confirm that a 16G puncture needle had entered the epidural space, and then the 25G spinal anesthesia trocar was inserted. A successful dural puncture was verified by the outflow of cerebrospinal fluid (CSF), 0.6% ropivacaine (1.5 ml 1% ropivacaine plus 1 ml 10% glucose) was injected. Then, spinal anesthesia trocar was pulled out, and an epidural catheter was inserted, and the catheter was placed cephalad to a depth of 3 cm. Additional anesthesia needed for epidural was determined according to the length of operation and the block plane of spinal anesthesia. CSEA was performed until it failed up to 5 times, and then it was replaced by general anesthesia.

### 2.3. Measurements

The patients characters, including age, height, weight, ASA grade, BMI were recorded. Primary outcomes were the success rate of first puncture attempt and average number of punctures. Secondary outcomes were the accuracy of landmark-guided positioning, location time, CSEA operation time, patient-satisfaction score, anesthesia characteristics, and complications of CSEA. The success of first puncture attempt was defined as needle entering skin, without changing puncture site and space, and the needle did not exit skin. In the condition that dural puncture was successful but without outflow of CSF, it is considered that the positioning is inaccurate and the puncture fails. The number of punctures included changing the puncture site or interspace, and reinsertion of the needle from the skin. The accuracy of the intervertebral space location is based on ultrasound-assisted positioning as the gold standard (100%), namely the overall agreement rate of ultrasound-assisted and landmark-guided L3-4 interspace. The time of ultrasound-assisted and landmark-guided positioning starts from touching the skin and ends when the positions of L4-5, L3-4, and L2-3 are marked. CSFA operation time is from the start of lidocaine local anesthesia to the completion of epidural catheterization. Patient satisfaction was scored using a 10-point numerical scale analog scale (1–10), with 10 being very satisfied and 1 being very dissatisfied. Complications of CSEA mainly count the incidence of hypotension/bradycardia, respiratory depression, nerve injury, epidural infection and headache. hypotension/bradycardia was defined as mean arterial pressure (MAP) < 65mm Hg or heart rate (HR) < 50/min. Respiratory depression was defined as the oxygen saturation (SPO_2_) < 90%.

### 2.4. Statistical analysis

The sample size was calculate using PASS v15 software. Based on our pilot study in obese patients, the first-attempt success rates with the ultrasound-assisted and landmark-guided were 81% and 53%, respectively. According to the calculation, 50 patients were required for each group at the 0.05 significance level (α = 0.05) and 90% power (β = 0.1). Considering possible dropouts, we increased the sample size to 59 patients per group. All data were performed using SPSS 25.0 or GraphPad Prism 8.0. Categorical variables such as the success rate of first attempt, anesthesia characteristics and complications of CSEA, were described as number and percentage, compared using the χ^2^ test or Fisher exact test. Student *t* test was administrated to compare the normally distributed data, which were presented as mean ± standard deviation (SD). Mann–Whitney *U* test was employed to compare non-normally distributed data, which were expressed as median and interquartile range (IQR). Statistical significance was set at *P* < .05.

## 3. Results

Overall, 118 women were recruited for the study from April 2021 to December 2021.After exclusion, 54 patients were randomized to ultrasound-assisted group (Group A) and traditional landmark-guided group (Group B). Three people in each group were excluded in the final analysis with the number of punctures was >5, and they were changed to general anesthesia (Fig. 1). Patient clinal characteristics were summarized in Table 1 and there was no significant difference.

**Table 1:** patient characteristics of both groups.

Group	Ultrasound-assisted (n = 51)	Landmark-guided (n = 51)	*P*
Age, yr	50.76 ± 3.89	51.29 ± 4.11	.168^a^
Hight (cm)	165.71 ± 12.67	164.12 ± 11.74	.261^a^
Weight (kg)	97.12 ± 21.62	93.46 ± 20.51	.127^a^
BMI, kg/m^2^	35.67 ± 2.16	34.72 ± 1.68	.376^a^
ASA grade			.401^b^
Grade II	36 (70.6%)	32 (62.7%)	
Grade III	15 (29.4%)	19 (37.3%)	

Values are expressed as mean ± standard division, or numbers (percentages).

ASA = American Society of Anesthesiologists, BMI = body mass index.

a Acquired from Student *t* test.

b Acquired from χ^2^ test.

The primary and secondary outcomes were shown in Table 2. The success rate of first attempt puncture in group A was significantly higher than that in group B (78.4% vs 52.9%, *P* = .007). Also, the total puncture numbers in group A were significantly less than that in group B (average rank 44.54 vs 58.46, *P* = .005). In terms of positioning accuracy, only 67% patients in group B achieved the correct location compared the presumed 100% rate in group A (*P* < .001). There was significant difference between the 2 groups in positioning time (313 seconds vs 206 seconds, *P* = .004) and CSEA operation time (378 seconds vs 489 seconds, *P* < .001). Patients in group A were more satisfied with the anesthesia compared with that in group B (average rank 76.76 vs 54.14, *P* = .002).

**Table 2:** Primary and secondary outcomes.

Group	Ultrasound-assisted (n = 51)	Landmark-guided (n = 51)	*P*
Primary outcomes			
Number of attempts			.005^d^
1	40 (78.4%)	27 (52.9%)	.007^b^
2	6 (11.8%)	10 (19.6%)	
3	3 (5.9%)	7 (13.7%)	
4	2 (3.9%)	4 (7.9%)	
5	0 (0.0%)	3 (5.9%)	
Secondary outcomes			
positioning accuracy	51 (100%)	34 (67%)	<.001^c^
Positioning time, seconds	313 [254–412]	206 [176–247]	.004^d^
CSEA operation time	378 [316–416]	489 [423–531]	<.001^d^
Patient satisfaction	76.76	54.14	.002^d^

Values are described as median (interquartile range), number (percentage), or average rank.

CSEA = combined spinal epidural anesthesia.

b Acquired from χ^2^ test.

c Acquired from Fisher exact test.

d Acquired from Mann–Whitney *U* test.

Anesthesia characteristics and complications were presented in Table 3. There was significant difference in the successful puncture interspace between groups (*P* = .039). The successful L3-4 puncture in group A were more than that in group B (41/51 vs 29/51, *P* = .02). The peak block plane of spinal anesthesia was no different between groups (*P* = .361). In addition, there were no significant difference between groups with regard to CSEA complications, including hypotension/bradycardia, respiratory depression, nerve stimulation, epidural infection, and headache (*P* = .858).

**Table 3:** Anesthesia characteristics and complications.

Group	Ultrasound-assisted (n = 51)	Landmark-guided (n = 51)	*P*
Successful puncture interspace			.039^c^
L3-4	41 (80.4%)	29 (56.9%)	.020^b^
L2-3	10 (19.6%)	19 (37.2%)	
L4-5	0 (0.0%)	2 (4.0%)	
L1-2	0 (0.0%)	1 (1.9%)	
Peak block plane of			
spinal Anesthesia			.361^c^
T6	19 (37.3%)	27 (52.9%)	.
T8	29 (56.9%)	22 (43.1%)	
≤ T10	3 (5.8%)	2 (4.0%)	
Complications of CSEA			858^c^
Hypotension, bradycardia	11 (21.6%)	13 (25.5%)	
Respiratory depression	1 (1.9%)	0 (0%)	
Nerve stimulation	1 (1.9%)	2 (4.0%)	
Epidural infection	0 (0%)	0 (0%)	
Headache	1 (1.9%)	2 (4%)	

Data are presented as numbers (percentages).

b Acquired from χ^2^ test.

c Acquired from Fisher exact test.

## 4. Discussion

This study indicated that for obese patients with CSEA, ultrasound-assisted positioning could improve the success rate of first puncture attempt and location accuracy, decrease total puncture numbers, and raise a higher satisfaction compared with the traditional landmark-guided location in open hysterectomy.

We chose to carry out this study in a secondary hospital in a Uyghur agglomeration area. The first reason is that due to geographical and dietary reasons, the proportion of obese patients is significantly higher in this area. The second reason is that due to the limitations of ultrasound technology, the current state of ultrasound application is unclear in secondary hospitals that still carry out a large number of CSEA. Previous studies have reported the application of ultrasound-assisted positioning in pregnant women and elderly patients who are predictably difficult to perform with CSEA, which indicate the promising application of ultrasound in CSEA.^[9,25]^ In addition, some studies hold the opposite opinion that use of ultrasound does not appear to increase the success rate of spinal anesthesia.^[26–28]^ Two distinct conclusions may relate to the quality of ultrasound, the proficiency of operator, or the types of patients they recruited.

This study showed that ultrasound-assisted positioning could better improve the success rate of first attempt (78.4% vs 52.9%, *P* = .007) and reduce the total number of punctures (average rank 44.54 vs 58.46, *P* = .005) in obese patients with open hysterectomy. This is consistent with previous reports that ultrasound improved the success rate of spinal anesthesia in patients with predicted difficult anatomy.^[29]^ In order to reduce the uncomfortable experience of anesthesia, we stipulate that the maximum number of punctures is 5 times. In this condition, 6 patients were replaced with general anesthesia due to the narrow puncture interspace, lack of cerebrospinal fluid outflow or other reasons. In terms of time consumption, ultrasound-assisted positioning takes more than that of traditional landmark-guided (313 seconds vs 206 seconds). In previous studies, ultrasound-assisted positioning time was shorter than our study.^[30,31]^ The underlying reason may be that twice scans of ultrasound was used in this study to locate the puncture interspace in these obese patients and operators varied in their proficiency of ultrasound. While, the time spent on CSEA in ultrasound-assisted group was less than that in landmark-guided positioning group (378 seconds vs 489 seconds, *P* < .001). Probably, precise positioning of ultrasound decreased the number of repeated punctures, and save more time for CSEA operations.

Another interesting conclusion that we have drawn from this study is that overall agreement rate of ultrasound-assisted and landmark-guided L3-4 was 67%. landmark-guided L3/4 was ultrasound-assisted L2/3 in 11/51 (21.6%) and ultrasound-assisted L4/5 in 6/51 (11.8%). Here we analysis the results of the first study comparing the concordance rate of L3-4 intervertebral estimated by ultrasound-assisted and landmark-guided in obese women. This conclusion has not been particularly concerned in previous studies, but we want to be clearly aware of the exact accuracy of traditional landmark-guided positioning in obese patients with the reason that accurate positioning is the premise for the safe administration of CSEA. Ultrasound-assisted positioning is inferior than computed tomography for accurate positioning with intervertebral spaces. However, the position of ultrasound-assisted was considered as the actual intervertebral space for the purpose of saving medical costs and operation time in this study. The result is convincing to illustrate that traditional landmark-guided positioning is not reliable enough in obese patients. Thus, there may be potential risks for some obese patients with traditional landmark-guided positioning. In this study, it happened 1 case (1/51) that successful puncture position was at L1-2 in landmark-guided group. Although it had brough no spinal cord injury to the patient, and it is enough to remind of the risk in incorrect positioning. CSEA is widely used in lower extremity and abdominal surgery, and is a better alternative to anesthesia for some patients with poor cardiopulmonary function who cannot tolerate general anesthesia.^[32,33]^ However, CSEA faces challenges in obese patients whose bony landmarks are not easy to touch.^[6,34]^ Traditional landmark-guided positioning takes longer time with the unclear and inaccurate puncture location, which increases the puncture attempts and brings poor anesthesia experience to the obese patient. Therefore, the application of ultrasound-assisted positioning may be a simple and fast way to improve the positioning accuracy.

Ultrasound application in CSEA is mainly divided into 2 forms, one is ultrasound-assisted with the other is real-time ultrasound-guided.^[35–37]^ Both ultrasound techniques have their own merits with regarding to operation manner, success rate, time costs and so on.^[9,38,39]^ In elderly patients with spinal anesthesia, real-time ultrasound-guided technique is not superior to the ultrasound-assisted since it has longer procedure time, lower satisfaction score, and is more difficult to perform.^[39]^ The requirements for real-time ultrasound technique are more proficient, which is difficult to be quickly grasped by anesthesiologists. Therefore, ultrasound-assisted technique may be more suitable to perform for anesthesiologists not skilled enough in using ultrasound. But it does not mean that real-time ultrasound-guided technique should not be carried out in CSEA. Although it takes more time, skilled real-time ultrasound-guided can improve the success rate of catheter placement, reduce puncture injury in some cases with anticipated anatomical difficulties, which was found beneficial especially for determining the correct needle insertion site and estimating the needle insertion depth of epidural space.^[29,38,40]^ Real-time ultrasound-guided technique may be more popular among anesthetists when obtain adequate education and training. And more advances in ultrasound technology will promote simpler operation and wide application in future.^[41]^ Anyway, it has to admit that ultrasonography has brought a revolutionary improvement in anesthesiology. And it is up to the anesthesiologist to choose the appropriate one according to the specific situation.

In traditional landmark-guided positioning, administration of CSEA relies primarily on the palpation of anatomical landmarks. This is a blind approach to the anesthesiologist and the variability of patient anatomy may lead to discomfort and complications occurring during CSEA operation.^[42,43]^ In consistent with this, we observed higher satisfaction scores with ultrasound-assisted compared with that of landmark-guided (average rank 76.76 vs 54.14, *P* = .002). Higher first success rate and fewer punctures result in less trauma during CSEA operation and higher satisfaction scores. However, in terms of CSEA complications, there was no significant difference between the 2 groups (*P* > .05). This shows that ultrasound-assisted positioning does not improve the safety of CSEA compare with that of landmark-guided. Among them, the incidence of hypotension/bradycardia was most, presumably from the high block level of spinal anesthesia. Other complications are less common, which may be due to the relatively small number of sample size. In terms of anesthesia characteristics, ultrasound-assisted CSEA is more likely to be successfully punctured at the L3-4 interspace (80.4% vs 56.9%, *P* = .02), which may be due to the accurate positioning, and the reduction of punctures, which avoided the need to replace interspace for next puncture. This is consistent with the previous conclusion.

This present study had some limitations. Firstly, it was a single-center study conducted in a secondary hospital. Secondly, the data collectors and performers were not blinded to group assignment due to the nature of this study. Thirdly, we did not perform a subgroup analysis on the obese patients, considering that the degree of obesity may be correlated with the difficulty in administration of CSEA.

In conclusion, administration CSEA with ultrasound-assisted positioning can improve the success rate of first puncture attempt, reduce the total puncture-numbers, increase the accuracy of location, and raise patients’ satisfaction in obese population. Given its convenience and simplicity in operation, administration of CSEA with ultrasound-assisted positioning is suitable for obese patients in secondary hospitals.

## Acknowledgments

Thanks to all the peer reviewers and editors for their valuable opinions and suggestions.

## Author contributions

**Data curation:** Haihong Yang, Huaqu Gong.

**Formal analysis:** Zuling Zhong, Yangyang Sun, Yinghai Liu.

**Funding acquisition:** Haihong Yang.

**Investigation:** Yangyang Sun, Yinghai Liu, Jingya Luo, Gu Gong, Yongjian Yang.

**Software:** Haihong Yang, Qin Zhang, Zuling Zhong, Lu Lin.

**Supervision:** Huaqu Gong.

**Validation:** Lu Lin.

**Writing – original draft:** Haihong Yang, Qin Zhang, Xuemei Dai.

**Writing – review & editing:** Gu Gong, Yongjian Yang.
